# Descemet Stripping Endothelial Keratoplasty in Microcornea for Pseudophakic Bullous Keratopathy With Anterior Chamber Intraocular Lens

**DOI:** 10.7759/cureus.19262

**Published:** 2021-11-04

**Authors:** Aanchal Singhal, Jaya Kaushik, Ankita Singh, Rakesh Shetty

**Affiliations:** 1 Ophthalmology, Armed Forces Medical College, Pune, IND

**Keywords:** congenital cataract, anterior chamber intraocular lens, pseudophakic bullous keratopathy, microcornea, descemet stripping endothelial keratoplasty

## Abstract

To report a case of bilateral pseudophakic bullous keratopathy (PBK) in a patient having bilateral microcornea with pre-existing anterior chamber intraocular lens (ACIOL) who underwent Descemet stripping endothelial keratoplasty (DSEK) with a successful postoperative visual outcome. A 36-year-old female, diagnosed with microcornea and congenital cataract in both eyes underwent lens aspiration sequentially followed by ACIOL implantation in both eyes. The patient reported to our centre and was diagnosed with bilateral PBK with ACIOL with microcornea. She also had associated secondary glaucoma, postoperative chronic uveitis, and hyphaema, which were controlled with medical management first and taken into consideration while planning DSEK. The patient underwent manual DSEK without intraocular lens exchange under local anaesthesia in both eyes sequentially with a good visual recovery postoperatively in both eyes. Descemet stripping automated endothelial keratoplasty (DSAEK)/DSEK seems a viable option in patients with microcornea who develop PBK following cataract surgery with retained ACIOL where there is absence of capsular support as well as deficiency of iris tissue.

## Introduction

Anterior chamber intraocular lenses (ACIOL) are commonly implanted in patients after cataract surgery with insufficient capsular bag support. However, they are associated with complications like endothelial decompensation leading to pseudophakic bullous keratopathy (PBK) (14%) [1], secondary glaucomas, and cystoid macular edema. Currently, the gold standard in the management of PBK is Descemet stripping endothelial keratoplasty (DSEK), Descemet stripping automated endothelial keratoplasty (DSAEK), or Descemet membrane endothelial keratoplasty (DMEK) [2]. Endothelial keratoplasty has largely replaced conventional penetrating keratoplasty for managing endothelial pathologies like aphakic bullous keratopathy and PBK [2].

DSEK/DSAEK in various types of retained intraocular lenses has been well described in the literature with varying success rates [3-5]. It has been revealed by multiple studies that DSEK is a relatively challenging procedure in presence of ACIOL due to reduced anterior chamber depth (ACD) and inherent complications associated with ACIOL [3-5]. Moreover, in the presence of conditions like microcornea, the surgery becomes an arduous challenge. Herein, we report an extremely rare case of a patient with bilateral PBK with microcornea and retained ACIOL who underwent DSEK with good visual outcome in spite of the extraordinary surgical challenge.

## Case presentation

A 36-year-old female presented to our centre with complaints of pain, redness, watering, photophobia, and diminution in vision both eyes for six months. She gave a history of prior lens aspiration and secondary ACIOL implantation in both eyes sequentially. On ophthalmic evaluation, she was found to have a distant best-corrected visual acuity (BCVA) of 6/36 in both eyes, corneal dimensions of horizontal diameter 9 mm and vertical diameter 8 mm, along with circumcorneal congestion, superficial vascularization inferonasally, epithelial and stromal edema, and multiple bullae in both eyes. The anterior chamber was quiet and ACD was 2.73 mm in the right eye and 2.72 mm in the left eye. Rest of the details could not be visualized. Digital intraocular pressure (IOP) was normal. Preoperative specular endothelial counts could not be measured due to corneal edema. Based on the above findings, patient was diagnosed as a case of PBK with ACIOL with microcornea in both eyes. She was managed medically first with topical steroids, sodium chloride solution, and anti-glaucoma medications. There were associated secondary glaucoma, postoperative chronic uveitis, and hyphaema, which were controlled with medical management first and taken into consideration while planning DSEK.

The patient underwent manual DSEK in the right eye, followed two months later by the left eye. Donor corneal endothelial counts were 2677 per mm^2^ in the right eye and 2823 per mm^2^ in the left eye. First, donor lenticule dissection was carried out with a 300-micron blade. In the recipient, descemetorrhexis was performed using a reverse Sinskey’s hook. A sheet glide was introduced into the AC through 4 mm superior limbal incision. A 6 mm trephine was used for donor cornea and the endothelial side marked. A cohesive viscoelastic was applied on endothelial side of the lenticule placed over the sheet glide and inserted into the anterior chamber using a cystotome. Subsequently, the incision was closed using three 10-0 monofilament nylon sutures and air was injected into the anterior chamber for apposition of donor lenticule to the host stroma. Corneal massaging was performed for the centration of donor graft. Since there was iris defect and probable lack of capsular support, air was maintained at least for 15 minutes before hydrating the wound. Numerous intraoperative challenges were faced due to congenitally small cornea, less ACD, and presence of ACIOL but were addressed by adopting certain modifications such as using sheet glide to avoid contact to ACIOL or angle structures and transplanting smaller donor graft for easier manoeuvring in the recipient eye.

Postoperatively, the patient was started on topical steroids, antibiotics, and lubricants. On the first post-operative day, donor lenticule was well apposed with host cornea and the intraocular lens was well centred. There was mild corneal edema which resolved gradually. Medications were slowly tapered as per the response. Thereafter, the patient was followed up daily for one week, then weekly for a month and, subsequently, monthly visits were continued for one year.

**Figure 1:**
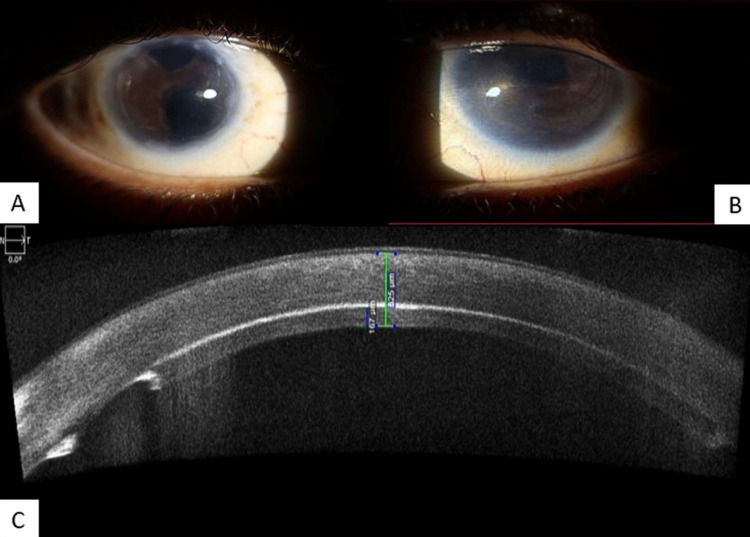
Slit-lamp images of both eyes and ASOCT of the left eye A, B: Post-operative slit-lamp images of both the clear microcorneas with lenticule well apposed and ACIOL in situ. C: Image of ASOCT showing well-adhered lenticule of the left eye ASOCT: Anterior segment optical coherence tomography; ACIOL: Anterior chamber intraocular lens

At the one year follow-up, she attained a distant BCVA of 6/12 in the right eye and 6/24 in the left eye. Anterior segment optical coherence tomography (ASOCT) revealed donor lenticules well adhered to host cornea with a thickness of approximately 153 µm in the right eye and 167 µm in the left eye. The cornea was clear and ACIOL was well centred as shown in Figure 1.

On specular microscopy, endothelial counts were 2543 per mm^2^ in the right eye and 2635 per mm^2^ in the left eye. ACD was 2.55 mm in the right eye and 2.45mm in the left eye. The IOP was 16- and 18-mm Hg with Goldmann applanation tonometry. Fundus examination revealed a healthy optic disc and macula in both eyes.

## Discussion

Endothelial keratoplasty in the case of PBK with retained ACIOL in itself is a herculean task, the presence of microcornea makes it even more challenging. This is due to the presence of ACIOL and smaller size of lenticule in microcornea, which makes centration of the lenticule difficult. Moreover, lesser ACD makes intraoperative surgical manoeuvring of the lenticule and its placement tricky. There were associated features of damage to angle structures along with corneal decompensation in our case leading to secondary glaucoma, postoperative chronic uveitis, and hyphaema, which were first managed medically and taken into consideration while planning DSEK.

There have been several studies in the past reporting the successful outcome of combined penetrating keratoplasty with ACIOL,iIris claw lenses, and transclerally fixated intraocular lenses [6]. However, only a few studies have reported the outcome of DSEK combined with different intraocular lens fixation techniques. In a case series of nine patients who underwent DSEK combined with a retro-pupillary fixated iris claw lens, stable endothelial cell counts and an improvement in visual acuity of 0.6 logarithm of the minimum angle of resolution (logMAR) units was reported at six months follow-up [7]. A case series of three patients who underwent femtosecond assisted DSAEK with fibrin glue-assisted sutureless posterior chamber intraocular lenses (PCIOL) has also reported an improved visual acuity post-surgery. In a similar fashion, a technique using glued scleral-fixated intraocular lenses (SFIOL) has shown lower endothelial cell loss compared to sutured SFIOLs as the securely fixed haptics in the sclera instead of the usage of sutures prevent a spring-like effect leading to pseudo-phacodonesis and provide a more stable configuration to the intraocular lenses [8]. Only a few studies have reported the outcome of DSEK in retained ACIOL. In a study of 11 patients who underwent DSEK in cases of PBK with retained ACIOL, stable endothelial cell counts and improved visual acuity was reported at follow-up [9].

Endothelial keratoplasty has now become the gold standard for the treatment of bullous keratopathy in presence of ACIOL [10]. Studies have shown that in cases of PBK with a well-positioned ACIOL and adequate ACD, DSEK may be a safe procedure with good endothelial survival that obviates the complications of intraocular lenses exchange [11]. To the best of our knowledge, there have been no reports describing the management of PBK in presence of retained ACIOL by DSEK in a case of bilateral microcornea with operated congenital cataract. 

Our case was unique and challenging as the surgeon had to face a multitude of surgical difficulties at every step. Also, the post-operative recovery was gradual due to the smaller size graft and early secondary glaucoma. The decision to continue ACIOL was taken since there was an already pre-existing iris chaffing and microcornea with a lack of posterior support. At the end of one year postoperatively, both the cornea were clear with a stable and well-centred ACIOL leading to a good visual outcome.

## Conclusions

DSEK seems a viable option in patients with microcornea who develop PBK following cataract surgery and ACIOL implantation where there is absence of capsular support as well as deficiency of iris tissue. It can be either manual DSEK or DSAEK. Other types of endothelial keratoplasty like DMEK are not very safe options in these cases as the chances of dislocation and loss of lenticule are very high.

Moreover, with secondary complications like secondary glaucoma, postoperative chronic uveitis, hyphaema, and inherent difficulties like pre-existing iris chaffing and microcornea with lack of posterior support in a patient with retained ACIOL obviate the need for intraocular lens exchange. Managing and taking into consideration these complications before planning a DSEK will help during intra-operative challenges. 

Therefore, in cases of PBK with a well-positioned ACIOL and adequate ACD, DSEK may be a safe procedure with good endothelial survival and good post-operative visual acuity can be achieved, without the need for intraocular lens exchange. 
